# The place and barriers of evidence based practice: knowledge and perceptions of medical, nursing and allied health practitioners in malaysia

**DOI:** 10.1186/1756-0500-3-279

**Published:** 2010-11-04

**Authors:** Nai Ming Lai, Cheong Lieng Teng, Ming Lee Lee

**Affiliations:** 1 Department of Paediatrics, Monash University Malaysia, JKR 1235, Bukit Azah, 80100, Johor Bahru, Johor, Malaysia; 2 Department of Family Medicine, International Medical University, Jalan Rasah, 70300, Seremban, Negeri Sembilan, Malaysia; 3 Clinical Research Centre, Hospital Tuanku Jaafar, Jalan Rasah, 70300, Seremban, Negeri Sembilan, Malaysia

## Abstract

**Background:**

Despite a recent increase in activities to promote evidence-based practice (EBP), it was unclear how Malaysian hospital practitioners received this new approach in medicine. This study examines their confidence and perceptions on EBP.

**Findings:**

We conducted cross-sectional surveys using a self-administered questionnaire during two EBP training courses in two Malaysian hospitals in January and June 2007. Our subjects (n = 144) were doctors and nursing and allied health staff (NAH) participating in the EBP courses. Our questionnaire covered three domains: confidence and understanding (six items), attitude (five items) and barriers to practice (four items). We presented simple descriptive statistics, including the sum ratings and the proportions with different responses for each item, and compared different groups using Mann-Whitney U test for scaled ratings and Chi-square test for dichotomous responses.

Ninety-two doctors and 52 NAH staff completed the surveys. Overall, doctors expressed slightly higher confidence on EBP compared to NAH staff. Out of a maximum sum rating of 27 over six items, doctors reported an average of 18.3 (SD 3.2) and NAH staff reported an average of 16.0 (SD 3.4), p = 0.002. Doctors were also more positive in their views on EBP. For example, 67.4% of doctors disagreed, but 61% of NAH staff agreed that "the importance of EBP in patient care is exaggerated", and 79.3% of doctors disagreed, but 46.2% of NAH staff agreed that "EBP is too tedious and impractical". Similar responses were observed for other items in the domain.

Doctors and NAH staff shared similar concerns on barriers to evidence-based practice. The highest proportions considered poor facilities to access evidence a barrier (76% of doctors and 90% of NAH), followed by poor awareness of evidence (62% of doctors and 70% of NAH) and time constraints (63% of doctors and 68% of NAH), p = 0.09 for the combined rating of four items in the domain.

**Conclusions:**

The findings of our survey suggest a need for greater efforts in promoting EBP among Malaysian hospital practitioners especially for NAH staff. From the responses based on the barriers to EBP, improving facilities for accessing evidence and promoting more user-friendly resources to address time constraints appear to be the priorities.

## Background

The belief and motivation of health practitioners are crucial in implementing evidence-based practice. Teaching motivated practitioners evidence-based practice (EBP) has been shown to improve their treatment decisions[1]. However, incorporating EBP into clinical practice presents many challenges, especially in developing countries[2]. Reports across various settings show different levels of receptiveness to EBP by health care providers, with the lack of support in terms of information technology (IT), local practice culture, as well as time constraints perceived as the major barriers to practising EBP [3,4].

In Malaysia, activities to promote EBP have increased in recent years. This followed the SEA-ORCHID (South East Asian Optimising Reproductive and Child Health Outcomes in Developing Countries) project, which was a collaborative initiative from 2003 that involved four South East Asian countries (Thailand, Malaysia, Philippines and Indonesia) to promote the synthesis and use of high quality evidence, especially on clinical problems relevant to this region [5]. The major activities of this project in Malaysia included local and national workshops on EBP for practitioners and trainers. Supported by the Australasian Cochrane Centre, a major emphasis of the project was to introduce Cochrane systematic reviews to clinicians and to encourage those interested to develop Cochrane systematic reviews. Authors of on-going reviews also received support through mentoring from experienced review authors and peers in dedicated sessions where review authors gathered and discussed their work. Under this project, there had also been exchanges between universities with the aim of strengthening their EBP curricula at the undergraduate and postgraduate levels for Health Sciences, including Medical, Nursing and Allied health curricula.

Despite the enthusiasm of trainers and practitioners who were involved in the activities, it was not clear how the hospital practitioners in Malaysia, including doctors, nurses and allied health staff saw this relatively new approach in medicine. Training in EBP may not be effective if the learners are not receptive to its concepts and approach. Success in implementing EBP also depends on how barriers identified by the practitioners are addressed by the decision makers. To assess the learners' confidence, attitude and their perception of barriers to EBP, we conducted a study on a group of hospital staff who attended our EBP courses, with the following objectives: We aimed to assess their levels of confidence and understanding on EBP, and their attitude by exploring their perception on the value of EBP in clinical practice, and assessed if there was any difference between doctors and other health care staff. Additionally, we aimed to evaluate how the major barriers to EBP in the local setting were as compared to what was reported in the literature.

## Methods

### Study design

We surveyed the participants using an anonymised, self-administered questionnaire.

### Subjects and settings

We recruited a convenience sample which consisted of the participants of introductory EBP courses in two Malaysian government hospitals: i). Hospital Batu Pahat, a district hospital in Batu Pahat, Johor, and ii). Hospital Tuanku Jaafar, a tertiary hospital in Seremban, Negeri Sembilan. The participants included doctors, nurses and allied health staff (NAH), who were representatives from various clinical departments and support services. The EBP courses were conducted in January 2007 in Hospital Batu Pahat, and in June 2007 in Hospital Tuanku Jaafar, Seremban.

The half-day courses provided an introduction to the concepts and approaches of EBP through short lectures, with exercises on formulating clinical questions and critical appraisal. The courses were organised under the initiative of the SEA-ORCHID project [5]. Speakers and facilitators of the courses received prior training under this project, which included training on EBP, Cochrane systematic review development and "Train the Trainer" workshop in both EBP and Cochrane reviewing. We conducted the surveys before the start of the courses.

### Questionnaire

Our questionnaire covered three domains: i). Confidence and understanding on EBP (six items), ii). Attitude: perceived value of EBP in clinical practice (five items), and iii). Perceived barriers to EBP (four items). We used a standard Likert scale for domains ii) and iii) with the following response options: "strongly disagree", "disagree", "unsure", "agree" and "strongly agree". A space was provided at the end of the questionnaire in which respondents were free to list barriers not covered in the questionnaire. We decided to phrase the questions negatively (e.g. "I find it hard to relate research findings with patient care") to reduce the participants' tendency to give socially desirable responses by choosing "agree" or "strongly agree" if the items were positively phrased (e.g. "I am able to relate research findings to patient care"), especially when the participants were in an EBP workshop. We avoided neutrally-phrased questions (e.g. "Relating research findings to patient care is....") because of the difficulty in standardizing the Likert-scaled responses for all items in the questionnaire".

We adapted the questionnaire from a version which was first drafted in June 2006 by one of the authors (LNM) to assess medical students[6]. During the initial validation, two authors (LNM and TCL), who were experienced teachers in EBP, determined the face validity of the items with reference from relevant literature on EBP [3,4,7-13]. We then piloted the questionnaire on a group of final-year medical students (n = 58) in August 2006. Internal consistency obtained from the pilot, expressed as Cronbach's alpha, was 0.60 (95% confidence interval for intraclass correlation coefficient: 0.43 to 0.74). Further revisions ensued in preparation for the current study, with several statements rephrased and two items removed, one (on the speed of tracking down an abstract) because it had a marked negative contribution to the overall internal consistency, and the other (on self-perceived competence in critical appraisal) as it was considered less suited to the respondents of these surveys, most of whom were naive to critical appraisal.

### Conduct of the survey

Two authors (LNM and LML) briefed the participants on the survey, including its aims, methods, approval status, and its anonymised and voluntary nature. Completion of the questionnaire was taken as consent. An administrative staff member not otherwise involved in the study collected the completed survey forms. The study was approved by the directors of the respective hospitals and was registered with the Malaysian National Medical Research Registry (NMRR).

### Analyses

For the six items relating to confidence and understanding on EBP, we accorded each response a rating in ascending order of confidence, from a minimum of one to the maximum rating depending on the number of options in the Likert scale. We reported the mean and standard deviation for each item. We also combined the ratings for all six items to form the sum rating, with a maximum sum rating of 27.

For items on perceptions on the value of EBP and barriers to its application, we assigned a rating for each response, as follows: One: "Strongly disagree", Two: "Disagree", Three: "Unsure", Four: "Agree" and Five: "Strongly agree". We combined the ratings for all items in each domain were to form the sum rating. As all statements were negatively phrased, lower ratings indicated more positive views and vice versa. Additionally, we categorised the responses into "Agree" (for responses that include "Agree" and "Strongly agree"), "Unsure" or "Disagree" (for responses that include "disagree" or "strongly disagree") in our statistical analyses to compare between the proportions of participants in these categories.

We used the following statistical tests: chi-square test for dichotomous data, and Mann-Whitney-U test to compare the sum ratings (SPSS 14 (Chicago, IL, USA)).

## Results

All 51 participants from Hospital Batu Pahat, and 93 from 150 participants (62%) from Hospital Tuanku Jaafar returned the questionnaires, making it a total of 144 respondents. Among the participants from Hospital Batu Pahat, 14 were doctors and 37 were NAH staff. From Hospital Tuanku Jaafar, 78 were doctors and 15 were NAH staff. Reliability analysis of all 15 items in the questionnaire yielded a Cronbach's alpha of 0.67 (95% CI of intraclass correlation coefficient: 0.54 to 0.77).

There were no significant differences in ratings when we compared the same category of staff between the two hospitals, i.e. doctors in Hospital Batu Pahat against doctors in Hospital Tuanku Jaafar, and similarly for NAH. Therefore, we only show the comparisons between doctors (n = 92) and NAH (n = 52) as a whole. The vast majority of the NAH were nurses (n = 43), and the remaining were allied health professionals including pharmacists, physiotherapists, dietitians and laboratory technicians. We did not separate each category of the allied health staff in our survey forms, as the workshops were catered mainly for medical and nursing staff, and we did not anticipate major attendance from the allied health staff.

### Confidence and understanding on EBP

**Figure 1:**
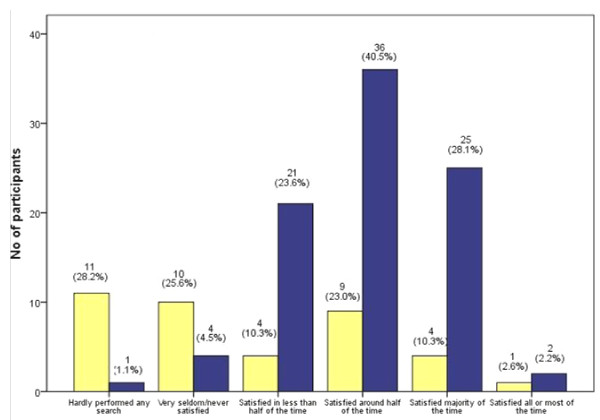
**Responses to item "How often are you satisfied with your search results?"**. The pair of bars on the extreme left indicates the proportions of participants who hardly performed any search, and the other pairs of bars illustrate the responses of the participants who had performed some literature search. Yellow square: Nurse and Allied Health (NAH). Blue square: Doctor.

Comparing the sum rating of all six items, doctors appeared slightly more confident on EBP than NAH staff. Out of a maximum score of 27, doctors had an average of 18.3 (SD 3.2) compared to 16.0 (SD 3.4) for NAH (p = 0.002). From Figure 1, the majority of the doctors (71%) were satisfied with their search results at least half of the time. On the other hand, over half of the NAH staff either hardly performed any search, or were seldom satisfied with their searches.

When asked to rate their own ability in understanding different parts of a paper, the respondents as a whole seemed more confident in reading the Introduction and Conclusion than the Methods and Results of a paper (Table 1). Doctors were more confident than NAH staff in understanding the Introduction and Conclusion of an article.

**Table 1:** Confidence on EBP: Ratings (out of four) on understanding different sections of an article

Items		Mean (SD)	p value
Understanding an article: Introduction	Doctors	3.2(0.7)	< 0.001
		
	NAH	2.7(0.6)	

Understanding an article: Methods	Doctors	2.7(0.7)	0.17
		
	NAH	2.5 (0.6)	

Understanding an article: Results	Doctors	2.9(0.7)	0.16
		
	NAH	2.7 (0.6)	

Understanding an article: Conclusions	Doctors	3.2(0.7)	0.01
		
	NAH	2.9 (0.7)	

**Figure 2:**
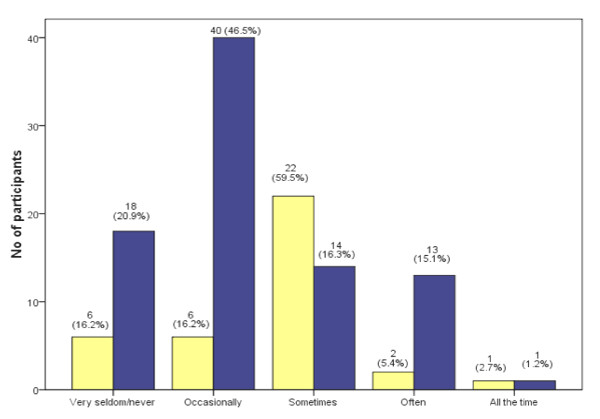
**Responses to item "How often can you tell a good study from a not-so-good one?"**. Yellow square: Nurse and Allied Health (NAH). Blue square: Doctor.

From Figure 2, only small proportions (16.3% of doctors and 8.1% of NAH staff) reported that they were able to differentiate a good study from a not-so-good one "often" or "all the time" (Figure 2).

### Attitude: perceived value of EBP in clinical practice

Table 2 shows a contrasting pattern of responses between the doctors and NAH. Overall, doctors were more positive than NAH staff, as the majority of the doctors disagreed or strongly disagreed, but the majority of NAH agreed or strongly agreed to all the negatively-phrased statements on the value of EBP. Consistently, more doctors than NAH staff disagreed or strongly disagreed on all the statements (p < 0.01 for all items). However, substantial proportions of both participant groups (from 14.1% to 31.7%) chose "unsure" as their responses to the statements.

**Table 2:** Participants' responses to each statement on the value of EBP in clinical practice.

Items		Percentage according to participant group				
		
		Strongly agree	Agree	Unsure	Disagree	Strongly disagree
Hard to relate research findings to patient care	Doctors	1.1	28.3	16.3	47.8	6.5
	
	NAH	7.5	42.5	22.5	27.5	0

The importance of EBP is exaggerated	Doctors	4.3	14.2	14.1	53.3	14.1
	
	NAH	7.3	53.7	22.0	14.6	2.4

EBP is too tedious and impractical	Doctors	1.1	2.2	17.4	63.0	16.3
	
	NAH	0	46.2	25.6	23.1	5.1

EBP is not feasible in this country	Doctors	3.3	23.4	15.6	44.4	13.3
	
	NAH	2.4	39.0	31.7	22.0	4.9

I value human views and experiences more than the evidence from research	Doctors	4.5	24.7	29.2	37.1	4.5
	
	NAH	2.5	57.5	22.5	12.5	5.0

### Perceived barriers to evidence based practice

Table 3 shows the participants' views on barriers to EBP. The majority agreed or strongly agreed on each barrier listed, with no significant difference between the responses of the doctors and NAH staff (p = 0.09 for sum rating of all four items).

**Table 3:** Participants' responses to each statement on perceived barrier to evidence based practice.

Items		Percentage according to participant group				
		
		Strongly agree	Agree	Unsure	Disagree	Strongly disagree
Practice norms and culture - lack of belief in EBP	Doctors	6.6	35.2	20.9	34.1	3.3
	
	NAH	2.4	56.1	34.2	7.3	0

Lack of awareness	Doctors	7.6	54.4	15.2	18.5	4.3
	
	NAH	7.5	62.5	20.0	10.0	0

Lack of time	Doctors	9.8	53.2	12.0	21.7	3.3
	
	NAH	7.3	61.0	12.2	17.1	2.4

Lack of good information technological support (e.g. computers with internet) in the wards	Doctors	40.2	35.9	7.6	13.0	3.3
	
	NAH	30	60.0	10.0	0	0

The largest proportion of the participants (76.1% of doctors and 90% of NAH staff) agreed or strongly agreed that a lack of good IT support at the point-of-care was a barrier. Remarkably, major proportion of medical and NAH staff (40.2% and 30% respectively) strongly agreed that this was a barrier, in contrast to all other items, in which only minor proportions of both the medical and NAH Staff chose "strongly agree" as their responses. Lower but similar proportions of medical and NAH staff noted a lack of time and a lack of awareness on EBP as barriers. Comparatively, smaller, but still significant numbers of medical and NAH staff alike considered unsupportive practice culture and a lack of belief in EBP in the workplace as barriers to EBP, as shown in Table 3.

## Discussion

This study offers a snapshot of how a group of Malaysian hospital practitioners perceive EBP. There have been similar surveys on Malaysian primary care doctors[14], private medical practitioners[10] and dental practitioners[15], but to our knowledge, this was the first study conducted on government hospital practitioners in Malaysia. Previous surveys in Malaysia showed that most medical or dental practitioners had not been exposed to formal EBP training, and it was likely that such training courses were organised regularly nationwide only in recent years under the SEA-ORCHID project [5]. Although surveys that measure self-perceived competence and attitude, like what we have undertaken here, have been criticized for not measuring the actual competence, we believe that self-perception and attitude play a crucial role in governing one's motivation to learn, practise and maintain a skill[16]. In our opinion, confidence and perception on EBP should be considered together with objectively measured EBP competence in evaluating overall success in promoting EBP. Here, we found that doctors were more confident and more positive in their perception of EBP compared to their NAH colleagues. However, both doctors and NAH had similar degree of concerns on the major barriers to EBP, with poor IT support at the point-of-care observed by most as a barrier, followed by time constraint and a lack of awareness to the importance of EBP.

We conducted the surveys at the beginning of the courses to avoid any possible influence from a motivational surge as a result of the EBP training. We believe that the pre-training responses would also reflect more closely the views of the hospital practitioners in similar settings around the country who had not been exposed to such training. It was notable that participants in our courses were mainly representatives from various hospital departments to fulfill the requirements for Continuing Medical Education (CME). The majority did not register voluntarily. It was therefore unlikely that our sample was biased towards motivated practitioners.

Differentiating a study of good quality from a study of lesser quality would require skills in critical appraisal, which seemed to be lacking in the majority of our participants, judging from their low confidence expressed on this item. This highlights an area of training need in future EBP workshops. However, as far as this study could assess from search satisfaction, understanding of journal article and the ability to tell a good study from a not-so-good study, we found Malaysian hospital doctors' self-reported EBP competence appeared to be comparable with that of the dental practitioners[15], and seemed higher than primary care doctors[14] and private practitioners in this country[10], although some differences in the survey tools and different periods of the studies precluded any direct comparison and meaningful conclusions. Specifically, earlier surveys in Malaysia either measured familiarity with guidelines[10], knowledge on basic EBP terms and literature search activities[14] and the perception of EBP on the respondents' care process and clinical decision-making[15], while our surveys focused on confidence and attitude on the value of EBP. Both our survey and the survey by Yusof et al [15] assessed barriers to EBP and showed similar results.

The comparatively negative attitude of NAH staff to EBP shown in this study might be due to their lower confidence. This might in turn be related to their level of qualification, as to-date, most nurses in Malaysia qualified through diploma courses. This finding suggests a need for greater efforts in promoting EBP to NAH staff. One possible way to achieve this is to increase the number of trainers with NAH background in EBP courses designed specifically for NAH staff[17]. At the undergraduate level, collaboration between health care disciplines in designing EBP curriculum may also bridge the gaps in confidence and perceptions in EBP between health care disciplines and facilitate team-working in future practice.

A vast majority of our participants agreed that poor IT support at the point-of-care, such as a lack of computer with reliable internet access, as well as a lack of time and poor awareness of EBP are barriers to implementing EBP. In particular, major proportions of the participants strongly felt that poor IT support was a barrier. This echoed the findings of earlier studies from Malaysia and other developing countries [10,14,18-20], and differed from the findings of studies conducted in developed countries, where institutional culture, rather than access to information or awareness seemed to be the major barrier[12,21-27]. Currently, inadequate infrastructure to access evidence in Malaysia is compounded by the limited availability and poor training of information specialists such as the medical librarians[28]. The findings highlight the priority for our policy makers when implementing EBP. While a reliable access to clinical evidence at the point-of-care may partly address the problem of time constraint, busy clinicians should also be directed to user-friendly, pre-appraised evidence-based resources for quick reference[29-31], and such resources, currently lacking in many institutions in the country, including the Ministry of Health Libraries[32], should be made widely available. Additionally, dedicated EBP training programmes for senior clinicians and policy makers may be the key to changing practice culture, as senior clinicians are often the role models for junior medical and NAH staff [1,7,33].

### Limitations

There are several limitations in our study. Although our questionnaire had better internal consistency (Cronbach's alpha) than the pilot version, it still fell short of the satisfactory level of 0.7[34]. Further revisions on the selection and wording of the items appear necessary. Specifically, we are not sure whether our negatively-phrased items could have introduced biases in the participants' responses, despite our justifications for adopting such format for the items. Next, our subjects, recruited from EBP courses in two hospitals, might not be representative of the hospital practitioners in Malaysia. The low response rate for the participants in Hospital Tuanku Jaafar further limited the generalisability of our findings.

## Conclusions

By exploring the perceptions towards EBP from a group of Malaysian hospital practitioners, our study highlights important issues to consider before implementing EBP in this country. Further studies should assess the effects of measures such as IT facilities and pre-appraised evidence based medicine resources at the point-of-care, incorporating relevant outcome measures such as objectively assessed EBP competence, clinical decision-making behaviour and patient outcomes.

## Competing interests

Non-financial: all three authors are motivated enthusiasts in EBP, and hoped the participants in their workshops would have a positive perception towards EBP.

## Authors' contributions

NML conceived and designed the study, developed the survey tool, oversaw the conduct of the study at Hospital Batu Pahat, collated, analysed and provided initial interpretation of the data, drafted the article and revised it critically for important intellectual content. CLT developed the survey tool, interpreted the data and revised the article critically for its important intellectual content. MLL oversaw the conduct of the study at Hospital Tuanku Jaafar, Seremban, entered and interpreted the data and provided input for the writing of the manuscript. All three authors have read and approved the final manuscript.
